# HBCUs and the Production of Doctors

**DOI:** 10.3934/publichealth.2017.6.579

**Published:** 2017-11-27

**Authors:** Marybeth Gasman, Tiffany Smith, Carmen Ye, Thai-Huy Nguyen

**Affiliations:** 1 University of Pennsylvania, 3819 Chestnut Street, St. Leonard's Court, Suite 140, Philadelphia, PA 19123, USA; 2 University of Michigan, 500 S. State Street, Ann Arbor, MI 48109, USA; 3 Seattle University, 90112th Avenue, Seattle, WA 98122-1090, USA

**Keywords:** black college, doctors, science

## Abstract

An important issue facing the world of medicine and health care is the field's lack of diversity, especially regarding African American doctors. African Americans made up 6% of all physicians in the U.S. in 2008, 6.9% of enrolled medical students in 2013 and 7.3% of all medical school applicants. The existing literature on the lack of diversity within the medical field emphasizes the role that inclusion would play in closing the health disparities among racial groups and the benefits acquired by African Americans through better patient-doctor interactions and further respect for cultural sensitivity. A large portion of current research regarding Black medical students and education focuses on why minority students do not go into medical school or complete their intended pre-med degrees. Common notions and conclusions are that many institutions do not properly prepare and support students, who despite drive and desire, may lack adequate high school preparation and may go through additional stress unlike their other peers. Historically Black Colleges and Universities (HBCUs) are institutions that were designed to support African American students by providing an educational learning environment that caters to their unique challenges and cultural understandings. Given that HBCUs have had much success in preparing minority students for STEM fields, and for medical school success more specifically, this article looks at the history of such universities in the context of medical education, their effective practices, the challenges faced by African Americans pursing medical education, and what they can do in the future to produce more Black doctors. We also highlight the work of Xavier University and Prairie View A&M University, institutions that regularly rank among the top two and top ten producers, respectively, of future African American doctors among colleges and universities.

## 1. Literature Review

### 1.1. History of Historically Black Medical Schools (HBMSs) and medical education

The demand for African American doctors is not a new dilemma. African American communities need and continue to need doctors that understand the unique cultural traditions, perspectives, and concerns of Blacks. Black medical schools have been integral to meeting this demand and Historically Black Colleges and Universities (HBCUs) have been instrumental in creating pipelines to these medical schools and others [1].

In 1910, there were only seven medical schools for African Americans and most were largely underfunded and deemed ill-equipped to provide a valuable education. At that time Abraham Flexner, an educator best known for his reform of medical education in the U.S., released a report that was a significant shift for medical education for African Americans. The “Flexner” report brought attention to the Black medical schools that existed, developed a limited perspective on their significance that was readily accepted, and provided ideas for improvements or suggested closings amongst the schools.

Before Flexner's report, there was little to no assessment of the Black medical schools that existed in the U.S. Of note, he described five out of the seven Black medical schools as being “in no position to make any contribution of value to the solution of the [Negro health] problem” and stated that they were “sending out undisciplined men, whose lack of real training is covered up by the imposing MD degree” [2]. These harsh conclusions were heavily weighted and eventually led to the closing of the five medical schools, leaving only Howard University School of Medicine and Meharry Medical School [2],[3]. Shortly afterwards, in the 1920s, the medical school admissions test (MCAT) was introduced as an admissions requirement. This test required a background in math in addition to science, which widened the achievement gaps in medical education due to lack of opportunities, and resulted in a decline in acceptance rates for African Americans in medical school [4].

Although most Black medical schools were closed, society did not deny their importance. That said, White medical educators and evaluators of medical schools only valued their significance when the education provided pertained to “hygienic principles” as a sanitarian. It was widely believed that Black doctors lacked the responsibility to fully care for their own people [2],[3]. It would not be until 1966, shortly after the Civil Rights Act, that African Americans were admitted into all U.S. medical schools. Prior to that time, Howard and Meharry medical schools were solely responsible for the production of Black doctors.

### 1.2. Difficulties of African American students in pre-medical education

Although there have been more opportunities and open doors for African American students to pursue a medical degree since the mid-1960s, many students leave the field and never make it into medical schools or fail to obtain a degree once in medical school. Current research on why it is difficult for African American students on the premed track attributes their struggles to a variety of reasons that range from lack of mentors to inadequate educational background to unsupportive academic environments.

Lack of mentors and social support is a recurring theme in literature about the success of African American pre-med students. In one study that examined the obstacles that racial and ethnic minority medical students face, participants stated that lack of support was a hindrance to their student success. Regarding family support, one participate commented that he would like to go home to talk about his medical studies to his father but “there is no way he will understand it” [5]. Likewise, in another study pertaining to what makes Black men successful in medical school and more likely to graduate, scholars found that exposure to medicine, medical practice, and social support from mentors were essential to those who successfully graduated from medical school [6]. Participants reported that Black men must act as role models for other Black men and attributed part of their success to the mentors in their own lives that served as a source of encouragement, motivation and confidence in their abilities.

In addition to the lack of social support minority medical students may face, they also often lack the academic background to effectively learn at their institution. For students who do not have a solid high school foundation in science, adapting to their science courses in college can be a challenge. Barr, Gonzalez, and Wanat conducted a longitudinal study on why there is generally an early decline in interest in premedical studies among underrepresented minority (URM) undergraduate students [7]. Through surveys and follow up interviews with 362 incoming Stanford freshmen, they found that the interest in becoming a physician among the students was influenced most profoundly by their high school and early college experiences in chemistry. If they struggled and did not have support in high school and during their freshman year of college, the impact was long lasting.

Unsupportive academic environments and institutional structures also play a vital role in the education of URM medical students. Problems pertaining to discrimination, prejudice, and racial identity are obstacles that African American students face and juggle in addition to their academic obligations [5],[8]. Stereotype threat, “where students underachieve due to negative, preconceived notions of their performance can be salient within a college environment, where deeply embedded societal stereotypes regarding intellectual competence are especially relevant” [8]. Some students may also attribute lack of support from faculty and administrators as discrimination [5]. If the institutions that African Americans students attend do not have the counseling and appropriate resources to help such students with these issues, they may not be able to academically perform to their full potential.

### 1.3. The role of HBCUs and HBMSs in the production of doctors

Rodriguez, Lopez, Campbell, and Dutton coined the term “HBCU Medical School Effect” to explain the concept that HBCUs have a significant impact on the overall rate of Black chairs, faculty, and students in medical schools [9]. Although HBCUs only represent 3% of all degree granting institutions in the nation, they represented 17% of the colleges supplying the most African American applicants to medical school in 2013. Xavier University and Howard University are also the top two producers of African American graduates of medical schools; their students accounted for 92 of the nation's African American graduating medical students, more than the top four Predominantly White Institutions (PWIs) that produce large numbers of African American medical school graduates combined [10].

Given HBCUs' success in educating and supporting future Black doctors, several researchers have examined the practices that have made them successful. In a study on premedical education at HBCUs, scholars found that HBCUs that devoted greater effort to premedical training, had larger proportions of biology and chemistry majors, stronger affiliations with medical schools, a range of externally sponsored enrichment programs, and boasted higher medical school acceptance rates than other HBCUs [11]. Out of the HBCUs in the study, those with the most acceptances for their students to medical school were very particular about allocating their resources to their premedical program and establishing relationships with institutions and programs for resources that they lacked.

Butler compared HBCU production of doctors today to the practices of Howard and Meharry in the 20th century [4]. She highlighted their successful interventions, such as having a core curriculum for pre-med students rather than options of course selection, tutors for all first and second year students, and beginning MCAT preparations during the first semester of freshman year. HBCUs are aware of the educational disparities that often exist between URM students and non-URM and do not hesitate to provide the guidance and support so that these students are acclimated as soon as possible. In a recent study, published in *Academic Medicine*, on the role of HBCUs, researchers found that “the strongest predictor of Black student enrollment is the number of African American department chairs at a particular school” [9]. Given that HBCUs are the only schools with a representative presence of African American department chairs and faculty, they have also been successful in producing future Black doctors because they are able to offer same-race role models for their students.

Scholars also suggest that HBCUs are particularly good at building strong pre-med and medical school pipeline partnerships and incorporating cultural competency skills and health disparities in their curricula [3],[9],[10],[12],[13].

## 2. Methods

To explore the work and impact of Xavier University of Louisiana and Prairie View A&M University, we conducted case studies at each institution over a three-year period. While conducting case study research, we interviewed 25 students, 7 faculty, and three administrators at each institution. The interviews were open-ended and lasted roughly 30–45 minutes. Those we interviewed signed consent forms, agreed to be recorded, and were provided with an interview protocol in advance of the interview. Of note, pseudonyms are used throughout the paper. After having the interviews transcribed, we read them as a team, with each person reviewing every transcript. We then coded the interview transcripts and noted important and reoccurring themes. In addition to the interviews, we also collected institutional data on student academic performance and did non-participant observations to triangulate our data.

## 3. Findings and Discussion

The next section of our paper explores two HBCUs that have important strengths in pre-medical education, Xavier University of Louisiana and Prairie View A&M University.

### 3.1. Xavier University of Louisiana, New Orleans, Louisiana

Xavier University of Louisiana is the only Historically Black College or University affiliated with the Roman Catholic Church in the country. Xavier contributes to the promotion of a more just and humane society by preparing its students to assume roles of leadership and service in a global society. The university's diverse learning and teaching environment incorporates research and community service throughout the curriculum. Xavier University has an enrollment of 2,976. Its first-year students have an average GPA of 3.37 and an average SAT combined math & verbal score of 985. Over 57% of the institution's first-year students are Pell Grant recipients, making them some of the lowest income students in the nation.

Xavier University has a formidable and nationally known history of producing doctors, which can be credited to Norman Francis, the institutions long-serving president [14]. In the 1970s, Francis read a report that caused him great concern — the report sounded the alarm that the number of Black doctors in the U.S. was dwindling at a steady pace. As a man of action, Francis decided to put his focus on becoming a major producer of Blacks in STEM and more specifically, Black doctors. Because of these consistent efforts over many decades, Xavier produces more Black students who apply to and graduate from medical school than any other college or university in the country [15].

During our campus visit to Xavier, we were struck by the generations of students that attended the institution for their education. Students told us of aunts, uncles, parents, even grandparents who had attended the school and recommended it to them. Unlike many HBCUs in the United States, Xavier has larger numbers of second generation college students from middle class families. As noted above, the institution still caters to many low-income and first generation students, but in the STEM fields, the dominant force of students came to college with college educated parents or close and influential relatives. Moreover, many of the students have various individuals throughout their families with STEM backgrounds. Students were also fully aware that attending Xavier meant that they were smart, committed, and would succeed in their given area. Those in the New Orleans area — from older generations working in the community to students at other colleges and universities in the area — were constantly given them this message.

Considered a leader in the STEM community, Xavier has two programs that have a significant impact on pre-medical education: its peer- and instructor-led drill system and its peer-led student tutoring centers. Both programs were developed for students enrolled in General and Organic Chemistry, courses that see high attrition among Black students. The peer- and instructor-led drill system monitors student progress as well as provides constant reinforcement of concepts and skills with two-hour drill classes once per week. Peer-led tutoring is an institutionalized practice at Xavier. Selected by faculty, peer tutors are available throughout the day at centers on campus, ensuring students have ample access to support.

From the point of view of students, if they attend the Xavier, they are confident and assured in their ability to become a doctor. They have no doubts about their future. Xavier's track record is well known among prospective students and is communicated with large billboards around the campus and throughout the surrounding states. African American communities know of Xavier's record of success in medicine. Of its nearly 3,000 undergraduate student body, 20% are pursuing degrees in chemistry, a subject matter critical to meet admissions requirements for medical and other health professional schools [16]. Students know that success has come before them and that they will be the demonstrated success for those in the future. Felecia, a sophomore shared her reasons for coming to Xavier, “I came to Xavier because I had a cousin who came, and then she told me how good they were with science programs, and that they were really geared towards getting people into medical school. I just felt like for me it was the best option to get myself into medical school”.

Students understand the history of Xavier and how this history will propel them to individual success. Another student, Dafina, explained it to us in this way,

One of the main driving points for me coming to Xavier was their success rate. Every time Xavier was mentioned, every time I say I go to Xavier University in Louisiana, people say oh you want to become a doctor, because they just know that Xavier University breeds doctors, successful doctors, all over the country. We are successful in every aspect, and every region, all over the world. So I know that, to me, that is what, every time I'm studying, I say okay, it's been done before, I can do this, and I just have to keep going.

Xavier's reputation, which as noted, is profound, but not unique in that many colleges and universities graduate large numbers of students who go on to be successful doctors. The difference is that Xavier enrolls ample numbers of African Americans — the institution bets on the potential success of these students whereas other institutions are not willing to take the same risks. As we mentioned earlier, Xavier's first-year students have an average GPA of 3.37 and an average SAT combined math & verbal score of 985. Moreover, 57 percent of the institution's first year students are Pell Grant recipients. These statistics are not the norm for pre-medical students at colleges and universities across the nation. In 2015, 31 percent of newly enrolled Black students in medical school came from homes with a total income of less than $50,000. This stands in stark contrast with just nine percent of White students in a similar social class (American Association of Medical Colleges, 2017). Xavier, with fewer resources, takes the risk and succeeds in a way that fosters future success and hope in the minds of African Americans.

The supportive environment at Xavier University came from the top. Recently retired President Norman Francis communicated the belief in every student's success to each student from the moment they stepped on campus. Students told us that during orientation, the president communicated a message of success that ran counter to what their friends at other institutions heard: “Doctor Francis spoke. And he said you know how most schools are like only 85% of you guys will graduate, everybody that you're sitting next to won't graduate, he said, no, we don't say that here, everybody will graduate at the end of the four years. So it's like you don't have a choice.” From the time, they step onto campus, students are constantly reminded of their ability to succeed. This message of success, however, is one that is framed around inclusivity. Anything else would be stamped out because for Black students in America, they have been surrounded by messages that question their presence in the school pipeline.

### 3.2. Prairie View A&M University, Prairie View, Texas

Prairie View A&M University (PVAMU) is the second oldest public institution of higher learning in Texas. The university has been recognized for its reputation of producing engineers, nurses, and educators. A member of the Texas A&M University System, the university is dedicated to fulfilling its land grant mission of achieving distinction in teaching, research, and service. Prairie View A&M University has an enrollment of 8,429 students. Its first-year students boast an average high school GPA of 3.10 and an average SAT combined math & verbal score of 845. Among the first-year students, 78% are recipients of Pell Grants, making it clear, that like Xavier — and even more profoundly, the institution educates the nation's low-income African American students.

During our visit to campus, we met families of students spanning several generations that had been taught by the faculty at Prairie View. Students learned of the institution and its track record in STEM and even particular faculty member's success with students from brothers and sisters as well as parents. Like Xavier University, the Prairie View students in STEM were often from middle-class families and had college educated parents. Of note, those students in the pre-medical program, in particular, were much more likely to come from college-educated parents and to have considerable exposure to STEM curricula and STEM role models. They regularly shared stories about their family member's experiences at Prairie View and how they were encouraged to attend and promised success.

PVAMU's Department of Biology has created two successful programs to improve the achievement and retention of their students in the STEM fields. Premedical Concepts Institute (PCI) is a rigorous ten-week summer program for incoming freshmen interested in pursuing STEM careers. The Cardiovascular and Microbial Research Center provides undergraduate students with research projects and mentoring that support independent problem solving.

One of the institution's approaches to ensuring that students see themselves as successful is to bring former students — now successful alumni serving as doctors across the nation — back to campus at the beginning of the academic year and throughout students' academic programs. Bernard, one of the Prairie View A&M University students, told us his professor ensures that students see success regularly and prominently,

He brings in people like doctors, dentists, veterinarians that are already making it and doing what we want to do. He brings them in and they motivate us, tell us what we need to do and they help us along. They're also African American so it helps as some people don't have that mindset that they can do whatever they want to do.

Prairie View Biology Professor Block says it best, “When you have a person who graduated from Prairie View and they come back and they tell you their journey, it makes a difference and you can see that they are successful, the Ph.D.s, the physicians, the dentists, the pharmacist, the podiatrist, the physical therapist, occupational therapist.” One of the Prairie View students, Jackie, enthusiastically affirmed professor Block's assertion with these words,

I've been in the situation where I was the only Black woman. So sometimes it's harder because some students may not understand you as well as far as your background or they may not be as receptive to listening to you and things that you have to say. So it does help. When you see people like you pushing forward through the certain struggles that they have to go through it pushes you to push even harder because you look at them and you're like okay you're going through this right now so I know I can push even harder because I'm not even in that situation.

Seeing demonstrated success not only matters to students, but serves as an impetus for future and sustained success.

Faculty members and faculty buy-in around promoting success is essential to student success as faculty members control the curriculum and day-to-day student interactions in STEM classes. The biology department at Prairie View A&M University has a pedigree of success. Families of students have attended the institution. Students often know before coming to the institution that they will be supported, uplifted, and believed in. They know because their cousins, parents, sisters and brothers have attended the institution and are a demonstration and manifestation of the success that is possible. Zoe, a student, explained to us with a sense of security in her mother's point of view and how it inspired her, “I chose Prairie View because my mom told me about it. I was looking at other schools. Southern Methodist University was my first choice and I had all other schools that I was looking at like bigger schools with more recognition like whenever you heard the name. But my mom told me that if you want to become a doctor you need to go to Prairie View. Prairie View is where people go to become doctors. That's why I decided to come to Prairie View”.

Most colleges and universities have a record of success in STEM, but fail to realize how important it is for that record to include racial and ethnic diversity. All too often, those leading pre-medical programs define success without attention to diversity, not realizing the long-term, negative impact this has on African American students' perceptions of belonging in a space that is dominated by White doctors. Even when established STEM programs have success among African Americans, they often fail to highlight this success or to keep in touch with graduates.

## 4. Conclusion

There is much to learn from HBCUs regardless of the type of college or university. First, having generations of families attend an institution is powerful as stories of success and concrete role models make a difference. This is particularly important for African Americans as witnessing and being surrounded by success has been shown to increase retention and persistence overall and in the field of medicine.

Second, HBCUs with strong ties to the community and a favorable reputation in local African American communities benefit through increased enrollment but also through the sustainability of interest and belief in self due. At both Xavier and Prairie View universities, pre-med students were uplifted by lore in the community around their talent and future success.

Third, both Xavier and Prairie View understand that a focus on chemistry along with exposure to medical culture provides the best foundation for medical school — a foundation that enables students to not only do well academically but to feel comfortable and confident about their inclusion in medical school.

Fourth, students interested in pursuing medical school benefit when the leadership of their undergraduate institution communicates messages of their success throughout the institution. These messages demonstrate a belief in African American success from the top, which spreads throughout the institution and is uplifting to students, especially as they tackle challenging curricula.

Fifth, the students at Xavier and Prairie View have been promised success by their faculty, relatives and members of the community. The belief in their success is evident in the curriculum, co-curriculum, and in the faculty mentoring that takes place on campus. Likewise, because many of the students are from families that have close relationships with Xavier and Prairie View and other HBCUs, they feel a kinship that is empowering to their academic and social success.

And lastly, Xavier and Prairie View, as well as most HBCUs, present an environment that is rich in same race role models. Thus, students are surrounded by success and see role models regularly, who send clear messages that African American success in science and in medical school is plausible and likely.

Although HBCUs in general have considerable success in their production of future Black doctors, the lack of diversity in the field is still a persistent problem. Continuing to create partnerships with medical schools and creating more opportunities for Black students will be pertinent to diversifying the medical field. This is work that needs to be done by all colleges and universities and not HBCUs alone. For too long, these institutions have been shouldering the work for which all higher education is responsible
